# Supplementary material for: Are carbon emissions trading and green financial instruments synergistic? -Comprehensive quantitative research based on content analysis
Source: PLoS One. 2024 Mar 7;19(3):e0298601. doi: 10.1371/journal.pone.0298601 (PMC10919601; doi:10.1371/journal.pone.0298601)
Supplement: S1 Data — (ZIP) [file pone.0298601.s001.zip › Data/地级市Z_F工作报告.zip!/╔╜╢1⁄2/╝├─╧╩╨/└┤╫╘╣1⁄2╓┌║┼í╛┬φ┐╦╩2╛▌═°í┐_2015.docx]

各位代表：

　　现在，我代表市人民政府向大会报告工作，请予审议，并请市政协各位委员提出意见。

　　一、2014年工作回顾

　　过去的一年，面对复杂严峻的国内外经济形势和紧迫繁重的改革发展任务,我们在中共山东省委、省政府和济南市委领导下，着力稳增长、促改革、调结构、惠民生、防风险，全力做好“加快科学发展、建设美丽泉城”各项工作，完成了全年经济社会发展主要目标任务。实现生产总值5770.6亿元，增长8.8%;地方公共财政收入543.1亿元，增长12.7%;固定资产投资3063.4亿元，增长16.1%;社会消费品零售总额2964.4亿元，增长12.6%;进出口总值105亿美元，增长9.6%。主要经济指标增幅均达到或超过全省平均水平。

　　（一）坚持市场导向，从影响经济社会发展的突出问题入手，积极稳妥推进各项改革，激发了市场活力和内生动力。按照市委、市政府《关于深入学习贯彻党的十八届三中全会精神全面深化改革的实施意见》，锐意进取，务求实效，全面深化改革实现了良好开局。

　　我们首先从政府自身改起，把加快转变职能、简政放权作为改革“先手棋”，政府机构改革基本完成，事业单位改革有序实施。推行行政权力清单制度，制定了权力清单目录和运行流程图。深化行政审批制度改革，调整减少各类行政许可、备案、确认项目66项，审批要件148项，取消或免征行政事业性收费35项，积极推行“两集中两到位”，已有15个部门的审批处(办)整建制进驻审批服务大厅。出台财税体制改革、金融体制改革和国有企业改革等实施意见，强化债务风险防控，调整财政支出结构，从严控制一般性支出，市级三公经费压减28%，腾出的资金主要用于改善民生、帮扶困难企业和贫困村庄。加快“政务云”建设，政府向社会购买服务的领域进一步拓宽。

　　我们努力为市场松绑、为企业添力。实行工商登记注册资本认缴制、先照后证登记制，全市新登记市场主体10万余户，增长68.9%。认真落实“营改增”、支持小微企业成长发展、出口退(免)税等政策，共为企业减负149亿元。放宽准入、强化服务，落实民营经济各项政策，民间投资增长23.1%，一大批民营企业加快发展壮大，齐鲁制药销售收入突破一百亿元。创新产业引导资金使用方式，推进科技金融创新发展，山东金融资产交易中心成立，挂牌额近400亿元，引导24家企业在“新三板”挂牌上市，居全省首位，济南农村商业银行开业，恒生银行济南分行筹建。市属44家国有困难企业帮扶解困和改革发展工作取得积极进展，解决了一批历史遗留难题，24家企业的职工得到一次性安置，部分企业走上振兴发展新路。农村土地承包经营权确权登记全面展开，启动农村产权市场体系建设，土地流转面积达到97万亩，有力促进了农村市场化进程。

　　（二）坚持以质量效益为中心，稳投资、调结构，拓市场、促开放，经济运行呈现稳中有进、稳中向好的积极态势。面对经济下行压力，我们突出重点、有扶有控，集中精力转方式调结构，实现了有质量的增长。税收比重达到79.9%，提高0.7个百分点;高新技术产业产值比重达到41.56%，提高1个百分点;服务业比重达到56.4%，提高1.1个百分点。

　　结构调整出现积极变化。传统产业改造提升加快推进，新材料、新医药、建筑产业化等新兴产业加速发展，规模以上工业增加值和利税分别增长10.1%和17.1%，提前完成“十二五”淘汰落后产能目标任务。信息软件、电子商务成长迅速，软件和信息服务业收入1750亿元、增长29.9%，电子商务交易额1608亿元、增长50.8%。全市金融机构本外币存贷款余额分别达到1.2万亿元和1万亿元。建成6个国家级科技园区、10个火炬计划特色产业基地，新认定35家市级以上企业技术中心。加快服务业提档升级，改造提升重点商圈，推进游客集散中心、方特东方神画、朱家峪、龙冈梦幻星空等一批文化旅游带动项目建设，全市亿元以上市场达到61个、年交易额近800亿元。

　　有效投资进一步扩大。项目建设三年行动计划圆满收官，681个项目完成投资2425亿元，在新兴产业、基础设施领域形成了一批有质量、可持续的投资项目。毫不动摇抓好招商引资工作，组织赴加拿大、美国、韩国等对外招商活动，引进市外投资851亿元，增长13.1%。开展对全国工商联重点企业系列招商活动，新签约项目72个、总投资2130亿元。22个重点片区施工面积达到4400万平方米，完成投资540亿元。济南创新谷、药谷、鑫茂齐鲁科技城等孵化器吸引了一批创新型企业入驻。10个省级以上开发区生产总值、固定资产投资、财政收入增幅均高于全市平均水平3个百分点以上。

　　开放型经济继续向好。进出口总值创历史新高，其中出口增长10.5%,扭转了两年来连续下滑局面。实际到账外资14.3亿美元，增长8.7%，融资租赁成为利用外资新亮点。服务外包离岸执行额23.9亿美元，增长58.3%。对外承包工程完成营业额21.5亿美元，增长9.3%。境外投资发展势头良好。济莱协作区建设扎实推进，启动城际铁路等重大基础设施规划建设，省会城市群经济圈一体化进程加快。

　　（三）坚持“六城联创”，实施重点攻坚，一批热点难点问题得到有效解决，提升了城市环境质量和生态宜居水平。围绕创城为民、创城惠民，统筹推进国家卫生城市、森林城市、水生态文明市、环保模范城市等“六城联创”工作，从广大人民群众反映突出的环境问题入手着力攻坚，推动美丽泉城建设取得新成效。

　　在全市人民大力支持和积极参与下，以治脏治乱为重点，强力推动露天烧烤、占道经营、“八小”行业、传染病防治等12项整治行动，完成了794个开放式小区、110处农贸市场、600多条背街小巷、546个城中村和城乡接合部的环境卫生整治，排查整治各类垃圾死角2174处，清运垃圾241万立方米，清除主次干道、居民小区的小广告、乱涂乱画140多万处，规范临时便民市场摊点1300余处，落实河长制、路长制等城市管理长效机制，市民工作生活环境更加便利舒适。

　　面对雾霾多发状况，我们积极应对，治标治本多管齐下，开展大气污染防治“十大行动”，狠抓燃煤、扬尘、工业生产和机动车排放等重污染源治理，启动东部老工业区搬迁改造和工业余热利用工程，大力推广太阳能光热利用与建筑一体化和先进节能减排技术，完成105蒸吨供热燃煤锅炉集中替代，淘汰黄标车40491辆，对3250处渣土处置工地和1700辆渣土运输车实行全时段监管，立案查处环境违法案件632件，大气质量综合改善率达15.9%。

　　加大治水、治山、治绿力度。水生态建设取得重要阶段性成果。开展防洪除涝、河道整治、水源连通、景观提升、湿地保护等系列工程，基本实现地表水、黄河水、长江水互连互通、联合调度，完成腊山河、大辛河等65条河道截污整治和绣江河、商中河、土屋峪等小流域综合治理，玉符河、龙脊河等河道综合整治工程加快实施，卧虎山、锦绣川等“五库连通”工程快速推进，全市集中污水处理率达95%。编制泉域重点强渗漏带规划，开展回灌补源工作，趵突泉群连续11年持续喷涌，泉水申遗工作进展顺利，成功举办第二届泉水节。我市荣获“中国温泉之都”称号。城区山体绿化提升、荒山绿化、水系绿化、绿色通道和农田林网等重点项目建设全面推进，启动千佛山风景名胜区、野生动物世界等公园景区建设提升十大工程，治理破损山体18处，建设牧牛山、卧牛山、卧虎山等山体公园13处，完成绿色通道绿化提升347公里，植树造林22.3万亩，新建绿地300万平方米，森林覆盖率达35.2%，城市绿化更加丰富多彩。

　　（四）坚持以基础设施建设和产业培育为先导，推进新型城镇化，加快城乡融合发展，县区经济实力持续增强。全市城镇化率达到66.4%。完成产业空间布局、新型城镇化和综合交通体系、济北新城、旧城更新等规划编制，开展新一轮中心城控制性规划修编。加快基础设施和公共服务向县域辐射延伸。济乐高速、机场路及南延工程、凤凰路、和平路等建成通车，济东高速、国道104线、220线和省道242线等改建工程以及二环西路南延、二环南路快速路和二环东路南延、纬十二路等重点工程进展顺利，新改建农村公路408.5公里，完成村级公路网化示范项目1220公里。新开辟和优化调整公交线路47条，城市道路通行能力提高20%。新建、改造水电气暖等各类管道管线400公里，改造完成玉函南北区等10个供水低压片区，唐冶热源厂建成运行，章丘电厂余热利用、腊山与西客站热源厂续建等工程加快推进，新增供热面积780万平方米。

　　扶持县区发展成效显著。圆满完成三年突破平阴、加快长清融入中心城区、支持济阳建设北跨产业发展区、继续帮扶商河跨越发展等目标任务，县域经济进一步壮大，县区财政收入实现三年翻番目标。中心城区服务功能持续增强，济南金融商务中心区、泉城特色风貌区、西客站片区、东部中央商务区等重点区域载体功能和文化品位不断提升，一批总部聚集区、特色街区、创意产业园区正在崛起，楼宇经济加快发展，已有税收亿元楼宇32座。推进产业北跨，集中启动了一批北跨发展项目,新材料产业园初具规模，鹊山龙湖、北湖、华山片区建设取得重要进展。南部山区绿色发展步伐加快，交通、环卫等基础设施不断完善，灵岩慧谷、柳埠旅游度假区等项目有序推进。

　　农业经济实现新发展。全面落实强农惠农政策，积极发展新型农业经营主体，现代农业示范园区达到200家、家庭农场958家，新认定市级农业龙头企业42家。现代种业、品牌农业、绿色生态循环农业和农产品精深加工业快速发展，农业技术、农业装备水平进一步提升，泉城农业公园成为省会现代农业展示的新窗口。蔬菜、肉蛋奶等产量稳定增加，粮食生产连续十二年丰收。

　　小城镇和美丽乡村建设稳步推进。刁镇、玉皇庙、万德、孔村等14个省市级示范镇完成投资10亿元，11个镇入选全国重点镇，新建续建农村新型社区44个，10.5万名农村居民入住新型社区。首批完成47个市级美丽乡村示范村和256个县级示范村建设，基本实现城乡环卫一体化全覆盖，农村人居环境持续改善。解决了20万农村居民和5.2万农村学校师生饮水安全问题。创新乡村扶贫解困工作，以选派100名“第一书记”驻村帮扶为抓手，探索启动了一批适合当地脱贫致富的好项目。

　　（五）坚持把社会效益放在首位，着力解难题、惠民生，创新社会治理，城乡居民生活进一步改善。在财政收支矛盾较大的情况下，我们尽力而为、兜住底线、保住基本、创新机制，推动社会事业蓬勃发展。城市居民人均可支配收入、农民人均纯收入分别增长8.7%和11.2%。居民消费价格涨幅为2.2%。全面完成为民办15件实事。

　　实施更加积极的就业政策。多渠道开发就业岗位，推动以创业带动就业，支持西街工坊、历下区大学生创业园等110家创业孵化基地开展创业培训、创业指导、创业孵化。新增城镇就业18.7万人，农村劳动力转移就业5.8万人。建立农民工公共服务五级网络和平台，促进了农民工更好地融入城市。

　　保障群众基本生活。连续第十次调整提高企业退休人员养老金标准，连续四年提高居民基础养老金标准，城乡低保、农村五保供养补助均有新的提高，完成新农合和城镇居民医疗保险制度整合，政府补助标准提高到每人每年320元。实施职工医保门诊统筹，公立医院综合改革实现县域全覆盖。社会化养老服务体系逐步建立，为80周岁以上低保老年人发放高龄津贴，“贴心一键通”入网用户达12.6万人。启动和续建了92个城中村改造项目，开工建设保障性安居工程29525套、提供可分配住房9218套，创建了具有济南特色的“六位一体”保障房管理模式，获得2014年中国人居环境范例奖。

　　社会事业健康发展。推进教育综合改革，加快现代职业教育体系建设，着力解决新建小区教育配套和外来务工人员子女入学难问题，新建中小学15所，新建、改扩建幼儿园112处，外来务工子弟学校达到82所。成功举办第三届非物质文化遗产博览会和山东省第五届文博会，文化惠民工程扎实推进，齐长城、城子崖遗址、老舍旧居等古迹保护力度加大，超额完成三年建设百座博物馆任务，建成各类博物馆、纪念馆等178座。全民健身活动蓬勃开展。稳妥落实“单独二孩”政策，人口自然增长率保持在6.5‰以内。

　　创新社会治理工作。以培育和践行社会主义核心价值观为主线，弘扬优秀传统文化，推进思想道德建设，文明城市创建工作扎实有效。建立社会组织孵化服务平台，新登记社会组织275家，家政网络服务平台年培训家政服务员万人以上。全市行政村和城市社区两委换届选举工作基本完成。开展净网行动，健全打防管控一体化机制，以大数据、云计算提升民生警务平台服务功能，平安济南建设取得新成效。组建统一、权威的食品药品监管机构，狠抓重点领域、重点环节、重点品种专项整治，保障群众饮食用药安全。便民放心菜工程实现全城覆盖。深入推进信访制度改革，创新信访积案化解方式，重点矛盾纠纷化解率达到92.5%。扎实开展重点行业、领域安全隐患排查整治工作，安全生产形势平稳向好。

　　在深入开展党的群众路线教育实践活动基础上，继续抓好中央八项规定等各项制度落实，加大行政监察和审计监督，推进党风廉政建设和反腐败斗争，严肃查处违纪违法案件，持之以恒纠正“四风”。实施党风政风行风民主评议，深化服务型政府建设，12345市民服务热线被确定为国家服务业标准化示范项目。深入推进依法行政,完善科学民主依法决策制度，推行政府法律顾问制度，自觉接受人大依法监督、政协民主监督、社会监督和新闻舆论监督，办结人大代表建议318件、政协委员提案710件，北跨议案办理取得重要进展。深入实施“六五”普法，健全城乡一体的法律服务体系。人防系统在全国军事斗争准备检验评估中荣居首位。统计、外事、侨台、民宗、慈善、老龄、残疾人、妇女儿童、红十字、社科、档案、史志、仲裁、防震减灾、双拥共建以及援藏援疆等工作都取得新的进步。

　　各位代表！过去一年的成绩来之不易，这是在省委、省政府和市委领导下，全市人民拼搏进取、艰苦奋斗的结果。我代表市人民政府，向全市各族人民、各界人士、驻济部队、武警官兵和中央、省驻济单位，向港澳台同胞、海外侨胞和国际友人，表示崇高敬意和诚挚感谢!

　　我们既要看到成绩，更要看到前进中的困难和挑战。投资增长乏力，新的消费热点不多，稳增长难度加大;传统产业改造升级任务艰巨，新兴产业、生产性服务业等新增长点尚处于培育阶段;经济发展方式比较粗放，创新能力不足，部分企业生产经营困难;县区发展不平衡，城区发展面临空间有限、产业培育滞后、发展后劲不强等情况，县域发展存在着基础薄弱、主导产业不突出、发展动力不足等问题;城市规划建设管理和环境治理还不能满足要求，雾霾频发、交通拥堵备受社会关注;民生工作与广大群众迫切愿望还有差距，安全生产领域尚存隐患;依法行政水平还需提升，抓落实解难题的韧劲不够、办法不多，解放思想、锐意进取的精神状态还需增强;少数政府机关工作人员不廉不勤、不作为、乱作为现象依然存在，腐败案件时有发生。我们要直面问题，先从政府自身找原因、抓整改，牢记使命，敢于担当，扎实有效解决问题、改进工作，决不辜负人民群众的重托。

　　二、2015年工作安排

　　2015年是全面深化改革的关键之年，是全面推进依法治国的开局之年，也是稳增长调结构的紧要之年。当前，我国经济发展进入新常态，新型工业化、信息化、城镇化、农业现代化持续推进，我国发展仍处于可以大有作为的重要战略机遇期。习近平总书记集中阐述了“四个全面”战略布局，既明确了奋斗目标任务，又提供了坚强保障。我们要把思想和行动统一到“四个全面”战略布局和中央对新常态的认识判断上来，按照山东省委“一个定位、三个提升”的要求，始终坚持发展第一要务，增强忧患意识，坚定必胜信念，埋头苦干，锐意进取，努力以新认识、新思路、新作为，争当转型发展、提质增效排头兵，开创省会改革发展新局面。

　　今年政府工作的总体要求是：全面贯彻落实党的十八大、十八届三中、四中全会和习近平总书记系列重要讲话精神，按照“四个全面”战略布局，坚持稳中求进工作总基调，主动适应和引领经济发展新常态，把转方式调结构和创新驱动放到更加重要的位置，着力提高经济发展质量和效益，努力稳定增长，狠抓改革攻坚，推进依法治市，统筹区域发展，加强生态建设，提升城市功能，着力保障民生，弘扬优秀文化，强化风险防控，全面做好“加快科学发展、建设美丽泉城”各项工作，努力完成“十二五”规划奋斗目标，促进经济平稳健康发展和社会和谐稳定。

　　今年全市经济社会发展主要预期目标是：生产总值增长8.5%左右，地方公共财政收入增长10%左右，固定资产投资增长15.5%左右，社会消费品零售总额增长12.5%左右。农业、服务业、规模以上工业增加值分别增长4%、8.5%和9%左右，高新技术产业产值比重提高1个百分点左右。进出口增长6%左右，实际到账外资增长5%左右。城镇和农村居民人均可支配收入分别增长8.5%和10%左右，居民消费价格涨幅控制在3%左右，城镇登记失业率控制在4%以内，人口自然增长率控制在7‰以内，完成省政府下达的年度节能减排和环境保护任务。以上指标，是按照“走在前列”的要求，结合济南实际提出的，必须经过艰苦努力才能实现。

　　为此要重点抓好以下工作：

　　(一)紧紧围绕法治政府建设，推进依法行政，狠抓改革攻坚，为经济社会持续健康发展保驾护航

　　全面深化改革，要靠法治来引领和推动。按照市委《关于贯彻落实党的十八届四中全会精神全面推进依法治市的意见》，用法治思维、法治方式破解深层次矛盾，以法治引领改革发展破障闯关、推动民生改善和社会进步，营造公开公正的法治环境和优质高效的发展环境。

　　坚持依法行政，更好履行政府职能。各级政府及其工作人员要牢固树立法治理念，带头尊法学法守法用法。坚持法定职责必须为、法无授权不可为，严格按照法定权限和程序履行职责、公正执法。健全科学、民主、依法决策机制和程序，完善决策问责和纠错制度，对重要投资项目、自然资源开发利用等重大行政决策实行目录管理。加强重点领域政府立法和规范性文件合法性审查。进一步完善政府法律顾问制度。健全行政执法程序和监督机制，加强罚缴分离和收支两条线管理，完善行政执法裁量权基准制度。科学构建市、县(市)区两级行政执法管理机制，稳妥推进重点领域跨部门、跨行业综合执法试点。完善行政复议制度和执法责任制，对国有资产监管、政府投资、政府采购等权力集中的部门和岗位实行分事行权、分岗设权、分级授权，加强内部流程监督。以预决算、公共资源配置、社会公益事业等领域为重点，全面推进政府信息公开。深入开展“直面问题，践行承诺”问题整改活动，完善政府部门公开评议述职制度。

　　继续简政放权，不断提高政府效能。制定公布政府权力清单、责任清单和市场准入负面清单，消除权力寻租空间，推进政府职能、权限、责任法治化。聚焦企业和群众反映强烈的问题，深化行政审批制度改革，继续取消、精简、下放一批行政审批事项，加强事中事后监管。推动向县区、园区、基层放权放责，推进区域综合评价评估、企业设立“三证合一”。加快各级政务服务中心建设，深化“两集中两到位”改革，实现“一个窗口受理、一站式审批、一条龙服务、一个窗口收费”，打造更加便捷高效公开的政务服务平台。加强服务效能建设，强化电子监察、社会监督和落实督查，防止不作为、慢作为，打通工作落实“最后一公里”。

　　全面深化改革，抓好改革措施落地。按照市委市政府深化改革总体部署，聚焦重要领域和关键环节，深化完善专项改革方案,明确时间表、路线图，落实督办责任，确保实现重点突破。深化预算管理制度改革，完善政府预算体系，规范政府债务管理。深化投融资体制改革，创新优化产业扶持资金管理方式，编制实施政府投资项目计划，推广政府与社会资本合作新模式。深化地方金融改革，健全金融、债务风险防控化解机制，确保不发生区域性、系统性风险。深化国企国资改革，完善法人治理结构，创新监管模式，鼓励发展混合所有制经济，提高国有资本利用效率，继续推进44户国有困难企业帮扶解困和改革发展。拓展民间资本投资领域，完善市场化价格形成机制。继续扩大政府向社会购买公共服务范围。做深做实重大政策、重大项目研究，扎实做好“十三五”规划编制工作。

　　（二）以创新引领产业转型升级，催生经济发展新动力，着力打造省会发展新高地

　　当前，推动我市经济稳定增长，既要强化增量带动，策划推动一批规模大、带动力强的重点项目，实施好“项目建设两年提升计划”;又要加快要素驱动向创新驱动转变，一手抓新兴产业培育发展，一手抓传统产业转型升级，锻造以人才、技术、品牌、服务为特征的省会发展新优势。

　　大力支持新技术、新模式、新业态、新产业发展。坚持高端引领，聚焦重点产业，主攻核心技术，及早布局、做大做强新材料、新信息、建筑产业化、节能环保等新产业，推出一批新项目、培育一批新企业、打造一批新产业链、形成一批新产业集聚区。巩固提升碳化硅、铌酸锂晶体材料等技术优势，尽快扩大规模、延展产业链条，培育壮大以石墨烯、纳米银浆等为核心产品的一批新兴企业，打造半导体、光电子、光伏等新产业基地。积极发展信息经济，用好国家电子商务示范城市和物流节点城市优势，大力发展电子商务、云计算、大数据、物联网等新业态，扩大在智能制造、研发设计、电子政务等领域的应用，努力建设“智慧泉城”。坚持培育和引进并举，加速推进建筑产业化发展，加快长清、章丘、济阳建筑产业化基地建设，引进培育一批知名建筑部品生产企业，拓展市场，集聚发展。加快节能环保产业园区建设，推动节能环保设备、新能源装备、绿色建筑、节能环保服务业等领域跨越发展。坚持质量强市和商标品牌培育战略，打造“济南智造”自主品牌。

　　推进传统产业向中高端迈进。借势“中国制造2025”和“互联网+”行动计划，加强信息技术与制造业融合互动，优化生产要素资源配置，推进产业链不断细分和不同产业链之间整合重组，改造提升钢铁、化工、建材等传统产业，培育发展产业新形态和新模式。围绕交通装备、机械装备、食品药品等优势产业，支持中国重汽等重点企业扩规提效、创新发展，策划建设相关零配件专业市场和配套企业集聚区。有序推进东部老工业区搬迁改造，力争2016年底前完成38家企业搬迁改造，积极发展工业旅游、现代物流、服务外包等产业，将老工业区打造成为高端产业集聚区和宜居宜业新城区。

　　助推民营经济发展壮大。切实落实加快民营经济发展的一系列政策措施，放宽市场准入，支持民间资本参与市政公用事业、基础设施等公共领域投资和国有企业改制重组，鼓励民间资本参与孵化器、加速器及特色园区建设。维护公平竞争的市场秩序，落实企业投资自主权，除法律规定外，由企业自行决定设计、施工、监理单位。完善市、县、园区三级中小企业服务网络，为中小企业提供融资担保、项目申报、法律维权等服务，推动个转企、小升规。落实好全国知名民企入鲁助推转调创洽谈会签约项目。加强企业家队伍建设，培育发展行业协会和商会，形成大力发展民营经济、尊重民营企业家的良好氛围。

　　集聚整合创新要素和资源。以济南高新区为龙头，加强创新载体建设，重点推进山东信息通信研究院、国家超算济南中心等重大源头创新平台，以及创新谷、金谷、药谷、新材料产业园等基地建设，启动创建国家自主创新示范区前期工作。落实我市《加快创建国家创新型城市的意见》和“科技十一条”，鼓励企业加强研发和承接转化先进适用科技成果，支持企业和高校、科研院所共建研发机构及产学研基地，推广山东工业技术研究院等产学研合作模式，让更多科研成果走出实验室，变成推动发展的现实生产力。提升科技服务功能，推进研发公共服务平台建设，促进科技与金融融合，加强金融信用担保体系建设，大力发展创业投资、天使基金，深入实施领军企业、“小巨人”、“金种子”计划，推动科技型中小微企业快速成长。全面实施知识产权战略纲要，提升知识产权保护和运用水平。大力推动齐鲁人才特区和留学人员创业园区建设，继续实施“5150”计划和高端外国专家项目，培养引进一批领军人才、拔尖人才和紧缺适用人才，发展壮大创新创业团队，让更多优秀人才圆梦济南。

　　（三）坚持城区经济和县域经济两翼驱动，优化经济发展空间格局，促进区域协调均衡发展。城区和县域是省会经济发展的两翼，必须发挥优势、统筹推进，让城区经济、县域经济两翼展翅齐飞，促动省会发展新跨越。

　　推动城区经济可持续发展。充分发挥城区要素集聚、产业集中、承载力强、贡献度高的优势，以楼宇、街区、片区、市场为载体，重点发展总部经济、楼宇经济、特色街经济，以及金融服务、设计研发等生产性服务业和新兴工业，推动产城融合。吸引大型企业集团、民营骨干企业将总部或区域性总部及研发、结算、销售(采购)中心等落户济南，打造一批总部经济集聚区，形成更多税收亿元楼。推进区域金融中心建设，大力发展普惠金融，加快企业股权、金融资产等综合性交易市场建设，支持更多企业上市、挂牌融资，推动以民间资本为主的地方金融组织有序发展。改造提升现有特色商业街区，重点策划打造一批新的国家级、省级文化旅游、商业休闲、创意设计特色街区。规范提升一批传统专业市场，启动国家级综合农产品物流中心建设，提升邮政、快递便民惠民服务水平，打造城市共同配送公共服务平台，促进住宿餐饮、批零贸易等生活性服务业转型升级。

　　做大做强县域经济。以园区为主要载体，强化招商选资和项目建设，补短板、培增量、抓两头、促中间，抓好24个中小产业集群发展，促进特色产业向主导产业转化，着力提升县域经济实力。进一步创新开发区管理、融资机制，完善基础设施和配套服务，节约集约利用土地，鼓励园区、企业建造多层厂房，引导有技术、有市场、成长性好的中小企业进驻标准厂房，促进人才、信息、资金、设备、技术等生产要素向园区集聚，加快制造业服务化，促进明水经济开发区等园区转型发展、争先进位。积极稳妥推进县域城镇化，以示范镇为龙头因地制宜发展特色镇域经济，打造一批工业强镇、农业重镇、文化旅游名镇。

　　深入推进南部绿色发展和北部跨河发展。扎实做好南部山区水源涵养、基础设施完善、乡村旅游、绿色产业培育等重点工作，稳妥推进南部生态经济区和生态新城镇建设。大力推动产业北跨，加快新材料产业园、鹊山龙湖度假区、济阳北跨产业发展区、商河经济开发区建设，加大对黄河以北地区重大基础设施建设扶持力度，统筹布局产业、居住和公共服务项目，提升北跨发展承载力。

　　（四）加强生态环境建设，提升城市功能品质，努力建设生态宜居的美丽泉城

　　以市民重大关切为导向，以“泉城特色突出、规划布局合理、建筑和谐美观、设施配套齐全、生态环境良好、管理井然有序”为目标，提高城市规划建设管理水平，实现城市品质提升和可持续发展。

　　为城乡发展提供规划保障。加快编制济莱协作区空间布局规划，推进53个片区控制性详规修编完善工作。坚持保护与发展并重，以古城和老商埠区复兴为核心，组织开展新一轮济南历史文化名城保护规划编制工作。认真落实旧城更新专项规划，有序推进81个旧城更新和棚户区改造项目，增加便民服务设施，进一步改善城市人居环境。

　　加强路、水、电、气、暖等基础设施建设。推进济青高铁、石济客专规划建设，实施国道104线改建工程，加快济东高速、二环西路南延等工程建设，力争年底实现二环南路全线通车。启动轨道交通R1线建设，加快R2、R3线设计立项，开展M线规划研究。实施宝华街、经一路延长线等一批城市主次干道建设改造。加快埝头湿地水源地和东区、雪山、旅游路、分水岭、官庄等水厂建设改造。加快城市雨污分流和老旧小区危旧管网改造，推动大金等污水处理厂建设，开展第二轮建成区污水直排口整治，提高污水处理水平。加快特高压工程和供电基础设施建设，加大局部区域电网和“一户一表”改造力度。优化城区天然气管网，推进东部应急调峰储配供应基地建设。加强综合管廊、油气管线、人防设施、应急避难场所等地下空间规划建设，开展地下管线普查和市政设施维护专项行动，加快建设数字城市地理信息系统，确保城市安全运行。

　　提升交通和城市管理服务水平。针对市民反映突出的交通拥堵问题，狠抓规划策划、路网改造、管理整治和“公交都市”建设。加快城市综合交通体系和交通改善系统、中运量公共交通等规划实施，推进新东站、火车站北场站和一批公交枢纽建设，提升西客站客运枢纽换乘能力。加强停车场建设管理，改善交通微循环，提高公交出行分担率，引导市民绿色低碳出行。完善“市、区、街、居”四级管理网络，以城市管理标准化推进城市管理精细化、长效化。深入开展全民爱国卫生运动和城乡环境综合整治，圆满完成国家卫生城市创建任务。

　　全力推进生态建设。坚决打好“治霾”攻坚战，实施大气污染防治“十大行动”，加大扬尘源头治理和管控，对钢铁、水泥、石化等高污染行业限期治理，加快工业余热利用和燃煤锅炉达标升级，扩大清洁能源推广使用，新增公交车70%以上、出租车100%使用新能源或清洁能源，完成改装淘汰9.2万辆黄标车、淘汰落后产能153万吨的任务。推进海绵城市建设，实现雨洪水科学利用。实施以“河湖连通、五水润城”为重点的水系治理，完成卧虎山、锦绣川等“五库连通”工程，推进玉符河综合整治及流域地表水转地下水工程，巩固提升护城河、小清河等河道生态化治理成果，加快湿地公园建设，多措并举节水保泉，整治修复满井泉等18处名泉，省控重点河流基本消除劣五类水体，完成国家水生态文明市试点任务。推进100个山体绿化工作，今年抓好27座破损山体治理、16处山体公园建设、10座山体绿化提升。加快天下第一泉景区、千佛山和英雄山景区改造提升，加大城市出入口、主次干道及节点、开放式小区、河道两侧绿化提升和环境综合整治力度，新增造林面积10万亩，基本消除城区裸露土地，达到生态园林城市绿化指标要求和国家森林城市检查验收标准。强化环保违法查处，在工业园区、环境公用设施等治污重点领域推行第三方治理，培育一批技术能力强、运营管理水平高的环保企业。通过标本兼治一系列措施的实施，让泉城再现更多的蓝天白云，彰显山、泉、湖、河、城相融相生的特色魅力。

　　（五）把“三农”工作作为重中之重，加快转变农业发展方式，促进农业现代化和农村改革发展

　　按照稳粮增产、提质增收、创新增效的要求，以全面深化农村改革为动力，加快转变农业发展方式，大力发展省会特色现代农业，让农业更强、农民更富、农村更美。

　　保障农产品有效供给与质量安全。以粮食生产功能区和现代农业园区为核心，优化现代农业产业布局，提高区域农业生产和管理水平，确保粮食和重要农产品供给。围绕60亿斤粮食产能工程，推进120万亩粮食高产创建功能区建设，力争粮食生产“十三连丰”。继续搞好农田水利基本建设，推进大中型灌区节水改造、小型农田水利重点县建设、山丘区水源工程、平原区坑塘整治、农业综合开发和土地综合整治，年内建设高标准农田11.2万亩。探索水利设施管护新模式，做好防汛抗旱工作，抓好农村饮水安全工程。深入实施农业科技创新和推广能力建设工程，组建科学研究、良种繁育、技术推广专业科技队伍。全面推开农产品质量安全监管工作，强化全过程监管体系建设。加快粮食产业园区建设，促进粮食产业转型升级。

　　加快转变农业发展方式。深入推进农业产业化，大力培育农业龙头企业，扶持农民合作社发展产后净化、分类分级、保鲜、包装等农产品产地初加工，引导工商资本搞好农产品精深加工、营销推广、品牌创立，加快农产品保鲜物流体系建设，促进一二三产业融合发展。突出发展蔬菜、畜牧、林果、花卉苗木等特色产业，抓好“一区六园”等现代农业园区建设，开展农产品品牌创建工作。积极培育新型农业经营主体，重点扶持发展30家农业龙头企业、30家示范合作社、30家示范性家庭农尝30家农业示范园区、30家蔬菜标准园和30家生态循环农业示范园，以农业经营体系创新加快农业现代化进程。

　　全面深化农村各项改革。坚持依法规范操作，基本完成农村土地承包经营权确权登记颁证工作。加快建设农村产权市场体系，设立济南市农业融资担保资金。开展农村集体资产改革，建立土地股份合作社、集体资产股份公司等新型集体经济组织，搞好资产经营，增加农民财产性收入。稳定农村土地承包关系，鼓励农民以出租、转包、入股、托管等形式流转承包经营权，发展多种形式适度规模经营。推进供销社综合改革试点，以强化基层社和创新联合社运行机制为重点，打造服务“三农”发展的生力军和综合平台。做好100个特困村帮扶工作，扎实开展精准扶贫，全面落实乡村扶贫解困“十项措施”，确保6万贫困农民增收脱贫、农村集体经济逐步壮大。

　　（六）坚持引进来走出去相结合，推动对内对外开放相互促进，争创新一轮开放型经济新优势

　　把握新常态下外向型经济新特点，研究制定《济南城市国际化行动纲要》，在更大范围集聚优质资源、拓展发展空间，推动新一轮对外开放，加快培育国际竞争新优势。

　　抓住中韩自由贸易协定签署的重大机遇，以建设中韩尖端产业合作济南先行示范区为突破口，与韩国重要城市建立合作伙伴关系，扩大产业协作和经贸往来。坚持出口导向，落实国家出口退税新政策，做好出口基地和示范区建设，推进机电、冶金等传统出口产业以及高端机械加工、生物医药、电子信息等科技型企业转型升级，培育出口竞争新优势，巩固传统国际市场。大力开拓新兴市场，用好国家信保政策，更多地组织企业参加国际展会，扩大外贸发展新增量。推进“海外济南”建设，鼓励指导企业开拓海外市场，积极参与境外上市、跨境并购、研发合作、对外承包工程和设立分支机构。围绕“一带一路”国家战略优化友城布局，推动更多企业走出去投资拓展。

　　把打造对外开放平台放在突出位置，推进济南航空城、齐鲁外包城、中外合作产业园等园区规划建设，着力吸引国内外服务外包龙头企业来济发展，配套做好中高端人才引进培养，加快建设服务外包示范城市，促进服务贸易发展壮大。进一步理顺综合保税区运营管理机制，加快核心区、配套区和进口商品保税展示交易中心规划建设，借鉴上海自贸区成熟经验，最大限度发挥综合保税区保税加工、保税物流、国际贸易、口岸通关四大功能，打造外向经济发展桥头堡。加快跨境电子商务平台建设应用，协调推动济南和青岛海关创新业务协作，实现区域通关一体化，提升贸易便利化水平。

　　实现招商引资新突破。准确把握先进地区与我市发展的阶段性差异和产业转移趋势，发挥济南科教、人才、区位、资源、产业基础等比较优势，盯住中关村等国内高端产业集聚区，加大力度定向招商，提高省会产业层次。顺应“互联网+”发展趋势，围绕国家三大支撑带发展战略和制造业升级十大重点领域，策划实施主题招商活动，引导市外资金参与我市产业转型升级和区域协调发展。按照省会城市群经济圈“一个高地”产业定位，错位招商，引导资金投向优势产业、新兴产业、现代服务业和总部经济，形成与周边城市产业协作配套的良性发展机制，打造带动区域融合发展的“服务型”和“创新型”增长极。

　　（七）挖掘利用好丰厚的文化资源，深化文化体制改革，推动文化事业和文化产业繁荣发展

　　济南历史悠久，文化灿烂。济南的先民们自古就在这片土地上刀耕火种、生息繁衍，孕育诞生了龙山文化、大舜文化、泉水文化，齐鲁文化在此交汇融合，形成了独特深厚的人文底蕴。我们要按照习近平总书记视察山东重要讲话精神要求，充分挖掘济南优秀传统文化，弘扬社会主义核心价值观，进一步丰富济南城市精神内涵，打造齐鲁文化发展新高地。

　　积极促进文化事业发展。完善公共文化服务体系，推进公共博物馆、纪念馆、爱国主义教育基地和文化馆、图书馆、美术馆免费开放，启动市博物馆新馆等文化设施建设，继续提升百座博物馆并纳入规范管理。扎实推进农村电影放映、农家书屋等文化惠民工程，扶持100个贫困村建设文化大院，建设50个村(社区)综合文化服务中心，打造40个基层群众文化活动示范点、40个规范化公共电子阅览室。开展全民阅读活动。深入实施文艺精品工程，引导创作具有民族风格、时代精神、地域特色的精品力作。推动历史文化名城复兴，加强齐长城、城子崖、东平陵故城、大辛庄遗址等文化遗产保护，改造提升一批老街、老宅、老院落等历史景观，开展“乡村记忆”工程，在美丽乡村建设中抓好各级示范村建设，保护好传统村落，更多地留住城乡的人文特色和历史记忆。推进第二轮地方志编修。高标准协助办好第22届国际历史科学大会这一首次在亚洲举办的历史学“奥林匹克”国际盛会。组织开展好“纪念世界反法西斯战争暨中国抗日战争胜利70周年”活动。

　　发展壮大文化产业。深化文化行政管理体制改革，打破条块分割格局，吸引社会资本投资兴办文化企业。引导国有文化经营单位加快建立现代企业制度。积极培育多层次文化产品和要素市场，支持文化企业上市或挂牌交易。依托英雄山文化市场和国家动漫产业基地等载体，吸引文化企业集聚发展。以创意设计、影视制作、出版发行、演艺娱乐、动漫游戏为重点，实施产业项目带动工程，促进文化与科技、信息融合，支持济南出版、济南演艺、舜网传媒等企业做大做强，培育一批骨干文化企业和文化品牌。积极开展对外文化交流，推动文化产品和文化服务走出去。加强文化执法，营造良好环境。

　　推动文化旅游融合发展。深入挖掘济南历史文化资源，在资源整合、策划包装、内涵提升上下功夫，打造“文化泉城”、“天下第一泉”、“百年商埠”等一批文化旅游品牌。推进方特东方神画、龙冈梦幻星空等项目开园运营，提升乡村旅游品质，吸附更多增量消费。编制全市文物古迹和景区景点地图，加快建设全市智慧旅游服务中心。精心筹办好第三届泉水节等重要节会，进一步提升“泉城济南”品牌影响力和竞争力。

　　（八）着力保障和改善民生，推动社会事业改革发展，更好地回应人民群众的新期盼

　　坚持守住底线、突出重点、完善制度，更加注重保障基本民生，更加关心低收入群众生活，更加努力保持社会大局和谐稳定。今年重点做好18件民生实事。

　　鼓励大众创新创业。实施就业优先战略，加强高校毕业生等重点群体就业帮扶，做好结构调整、过剩产能化解中失业人员的再就业工作。完善创业融资扶持体系，筹建我市首所创业大学，推进县(市)区创业学院建设，抓好省市创业型街道社区创建，培育推出更多众创空间。完善农民工综合服务五级网络，为农民工就业、培训、权益保障提供优质服务。健全劳动用工机制，加强劳动保障监察执法。全市高校毕业生总体就业率稳定在85%以上，援助就业困难人员应扶尽扶，新增农业富余劳动力转移就业5.2万人，新增城镇就业10万人。

　　着力提升保障水平。统筹考虑城乡社会保障一体化发展，完善城乡居民基本养老保险制度和基本医疗保险制度，完善落实被征地农民基本养老保障政策。实施机关事业单位养老和医疗保险制度改革。城乡居民基础养老金最低标准由每人每月75元提高到85元。城乡居民医保财政补助标准由每人每年320元提高到380元。基本公共卫生服务经费财政补助标准由人均35元提高到40元。继续做好城乡低保和企业退休人员养老金调整工作。加快公共租赁住房、城中村和棚户区改造等保障性安居工程建设，其中新开工公共租赁住房4400套。推进社会救助体系建设，健全留守儿童、残疾人等重点人群权益保障体系，实施医疗救助“一站式”结算，重点救助对象结算比例提高到70%。抓好养老服务体系建设，培育发展养老产业，促进医疗、养老、保健等多行业融合发展。

　　大力发展社会事业。深化教育领域综合改革,推动更多中小学提高教育教学质量，减轻学生课业负担，让孩子们有更多时间认识自然、发展兴趣、增强体魄。完善现代职业教育体系，发展一批急需的特色专业、特色学科、特色学校,培养更多技能型、实用型人才。推进公共卫生服务均等化，加快公立医院改革，发展社会办医和医师多点执业，深入实施“智慧健康”工程，提高人口优生优育水平，创建健康城市。盘活用好体育场馆资源，推广全民健身运动，做大做强体育产业。

　　深化社会治理创新。按照“政府扶持、社会运营、专业发展、项目合作”的思路，建立以社区为平台、社会组织为载体、专业人才队伍为支撑的社会服务管理新机制。培育一批基层组织和社会组织，承接更多政府公共服务职能。充分发挥12345市民服务热线作用，加快网格化管理全覆盖，推动社会服务管理向基层末梢延伸。抓好村(居)委会换届选举收尾工作。积极创建国家食品安全城市，提升食品安全监管水平。从严抓好安全生产和人员密集场所安全管理工作，坚决遏制重特大安全生产事故发生。深化平安济南建设，完善立体化社会治安防控体系，依法防范和惩治各类违法犯罪活动。加强普法宣传工作，推进覆盖城乡居民的公共法律服务体系建设。健全有效预防和化解社会矛盾机制，提高处置公共安全危机和突发事件的能力，切实维护社会和谐稳定。深化文明城市、文明行业、文明单位、文明社区、文明村镇创建活动，推动精神文明创建工作常态化，不断提升市民文明素质和城市文明程度。加强国防和民兵预备役工作，推进军民融合深度发展，争创双拥模范城“八连冠”。继续做好民宗、人防、侨务、对台、仲裁、地震、气象、档案等工作。

　　各位代表，作风建设永远在路上。我们将巩固和拓展党的群众路线教育实践活动成果，牢固树立群众观点，持之以恒纠正“四风”，认真遵守政治纪律和政治规矩，全面加强反腐倡廉制度建设，坚持党风廉政建设与经济社会发展一起谋划、一起部署、一起考核，健全惩治和预防腐败体系，加大财政预决算、重大项目审计力度，严格领导干部经济责任审计，推进公车改革，所有公务人员和一切公务活动都要厉行节约、反对浪费，切实做到廉洁勤政。我们将自觉接受市人大及其常委会法律监督、工作监督和市政协民主监督，严格执行市人大决议决定，认真办理人大代表建议和政协委员提案，广泛听取民主党派、工商联、无党派人士、各人民团体意见，高度重视社会监督和新闻舆论监督。我们将鼓足干劲，干事创业，通过一件件小事的办理，一件件实事的落实，一件件难事的解决，为人民群众谋求更大福祉，赢得更多信任和支持。

　　各位代表，我们进入了省会改革发展的新时期。新常态彰显新趋势，新征程孕育新希望。让我们紧密团结在以习近平同志为总书记的党中央周围，在省委、省政府和市委领导下，与全市人民携手并肩，攻坚克难，全面完成“十二五”规划各项任务，努力在全面建成小康社会进程中走在前列，为加快建设美丽泉城、实现人民群众新期待做出新的业绩！
